# Acceptance and Usability of an Innovative mDentistry eHygiene Model Amid the COVID-19 Pandemic Within the US National Dental Practice-Based Research Network: Mixed Methods Study

**DOI:** 10.2196/45418

**Published:** 2023-08-18

**Authors:** Jin Xiao, Dorota Kopycka-Kedzierawski, Patricia Ragusa, Luis Alberto Mendez Chagoya, Kimberly Funkhouser, Tamara Lischka, Tong Tong Wu, Kevin Fiscella, Kumari Saswati Kar, Nisreen Al Jallad, Noha Rashwan, Johana Ren, Cyril Meyerowitz, National Dental Practice-Based Research Network (PBRN) Collaborative Group

**Affiliations:** 1 Eastman Institute for Oral Health University of Rochester Rochester, NY United States; 2 Kaiser Permanente Center for Health Research Portland, OR United States; 3 Department of Biostatistics and Computational Biology University of Rochester Rochester, NY United States; 4 Department of Family Medicine University of Rochester Rochester, NY United States; 5 River Campus University of Rochester Rochester, NY United States; 6 School of Dentistry University of Alabama at Birmingham Birmingham, AL United States

**Keywords:** teledentistry, mDentistry, oral diseases, virtual visit, intraoral camera, COVID-19, pandemic response, mobile phone

## Abstract

**Background:**

Amid the COVID-19 pandemic and other possible future infectious disease pandemics, dentistry needs to consider modified dental examination regimens that render quality care and ensure the safety of patients and dental health care personnel (DHCP).

**Objective:**

This study aims to assess the acceptance and usability of an innovative mDentistry eHygiene model amid the COVID-19 pandemic.

**Methods:**

This pilot study used a 2-stage implementation design to assess 2 critical components of an innovative mDentistry eHygiene model: virtual hygiene examination (eHygiene) and patient self-taken intraoral images (SELFIE), within the National Dental Practice-Based Research Network. Mixed methods (quantitative and qualitative) were used to assess the acceptance and usability of the eHygiene model.

**Results:**

A total of 85 patients and 18 DHCP participated in the study. Overall, the eHygiene model was well accepted by patients (System Usability Scale [SUS] score: mean 70.0, SD 23.7) and moderately accepted by dentists (SUS score: mean 51.3, SD 15.9) and hygienists (SUS score: mean 57.1, SD 23.8). Dentists and patients had good communication during the eHygiene examination, as assessed using the Dentist-Patient Communication scale. In the SELFIE session, patients completed tasks with minimum challenges and obtained diagnostic intraoral photos. Patients and DHCP suggested that although eHygiene has the potential to improve oral health care services, it should be used selectively depending on patients’ conditions.

**Conclusions:**

The study results showed promise for the 2 components of the eHygiene model. eHygiene offers a complementary modality for oral health data collection and examination in dental offices, which would be particularly useful during an infectious disease outbreak. In addition, patients being able to capture critical oral health data in their home could facilitate dental treatment triage and oral health self-monitoring and potentially trigger oral health–promoting behaviors.

## Introduction

### Background

Amid the COVID-19 outbreak, dental health care personnel (DHCP) are at a high risk of contracting SARS-CoV-2 because of the close physical proximity between the DHCP and their patients and the absence of enhanced levels of personal protective equipment (PPE) [1-3]. Traditional dental examination relies on person-to-person examination, which poses tremendous challenges during the infectious disease outbreak for reasons including but not limited to infection control, exhaustion of PPEs, chairside time management, and treatment compliance. Although dentistry has been practiced for years using person-to-person visual and tactile intraoral examination now more than ever, dentistry should consider augmenting existing practices with virtual dental services involving a wide variety of technologies and tactics.

Supplementing traditional dental examinations (eg, comprehensive and hygiene recall examinations) with virtual examinations could potentially reduce the exposure risk for patients and DHCPs and preserve a large volume of PPEs that may be in short supply during a pandemic. In the current dental examination model, using the hygiene examination as an example, a single hygiene examination consumes 2 PPEs for the dentist alone because of the need to change PPEs between the dentist’s chairside patient and hygiene examination patient [4]. Traditional hygiene examinations also increase the challenge of infection control because of frequent switching of PPEs and dentists running between dental operatories [4]. In the era of the COVID-19 pandemic, dentistry would benefit from modifying dental examination regimens that render quality care and ensure the safety of patients and DHCP, especially the hygiene examinations.

### Objectives

In this digital era, our long-term goal is to develop an innovative mDentistry model (mDent) [5-7]. The mDent leverages the advantages of virtual dental visits and digital mobile health (mHealth) tools, such as intraoral cameras, to deliver virtual oral examinations, treatment planning, and interactive oral health management on a broad population basis [5]. In the mDent model, patients capture intraoral pictures at home before visiting the dentist. Capable patients could perform this independently by watching a photo-taking tutorial video, reducing DHCP instruction time during a virtual visit. The DHCP could assess dental health from intraoral pictures. The dental hygienist would take intraoral x-rays and additional intraoral pictures capturing critical soft and hard tissue in the oral cavity during the hygiene visit. After a convenient virtual dental visit with the dentist to examine findings and treatment plans, patients will have an in-office visit to confirm the findings of the virtual examinations and receive a definite dental treatment, if needed. The conversion of the traditional dental examinations to mDent virtual examinations builds upon the diagnostic reliability of teledentistry [8-10] and the rapid advancement of mHealth tool use by all-age Americans [11,12]. Clinicians have been using intraoral photos [8-10] and live video [13,14] to diagnose caries and predict with high accuracy the appropriate treatment modality for pediatric patients. However, 2 critical components of the mDent model—virtual hygiene examination (eHygiene) and patient self-taking intraoral images (SELFIE)—have not been previously evaluated.

Therefore, in this mDent eHygiene study, we piloted both eHygiene and SELFIE components within the National Dental Practice-Based Research Network (PBRN) [15]. We aimed to assess the feasibility and acceptability of implementing mDent eHygiene while exploring the ability of patients to take internal photos using health tools in their home setting.

## Methods

### Overall Study Design

**Figure 1:**
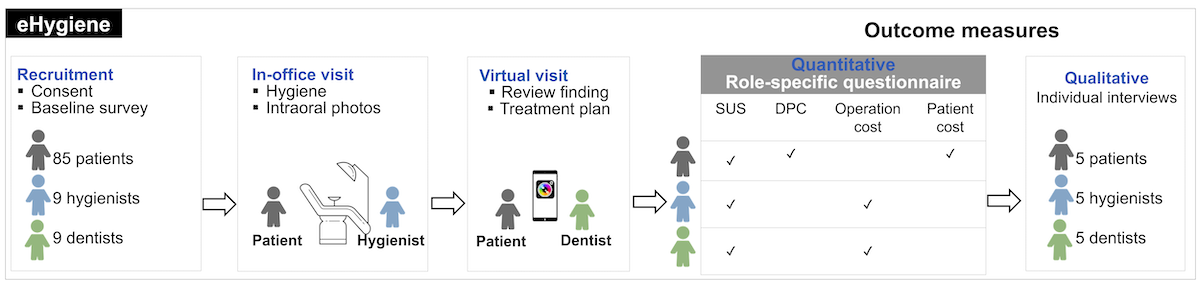
Specific aims, study design, and outcome measures. SUS: System Usability Score; DPC: Dentist-Patient Communication.

This study used a 2-stage implementation investigation to assess the acceptance of 2 components (eHygiene and SELFIE) of the mDent eHygiene model among patients and DHCP (dentists and dental hygienists who were members of the National Dental PBRN). This mDent eHygiene study used mixed methods (quantitative and qualitative) to collect outcome measures and conduct data analysis. Our study protocol has been detailed previously [7]. The study flow is shown in Figure 1.

Briefly, the first stage, the eHygiene session, was designed to assess the acceptance of and barriers to mDent eHygiene among patients and DHCP. We enrolled 85 adult patients and 24 DHCP (12 dentists and 12 hygienists) from 12 dental practices in the Northeast region of the National Dental PBRN. The hygienist at each participating practice enrolled approximately 12 (6-15) hygiene recall patients. A 20-minute instructional video for taking intraoral images was provided to the participating hygienists for training purposes. Patients received 1 in-office hygiene visit to collect the required clinical parameters and intraoral images with the hygienist. These patients then received 1 virtual visit with the dentist to review examination findings and treatment plans.

The second stage, the SELFIE session, was designed to assess the patient’s capability to generate intraoral images using mHealth tools. Hygienists invited one of their patients who completed the first stage eHygiene session to participate in the SELFIE session. In total, 4 patients volunteered to participate in the self-taken intraoral photo session under virtual guidance from the same hygienist during the eHygiene stage.

### Outcome Measures and Statistical Analysis

#### System Usability Scale

The System Usability Scale (SUS) instrument was used to assess the acceptance of the mDent eHygiene approach. The SUS instrument [16-18] is widely adopted in business and technology industries and mHealth fields to measure and quantify the perception of product and service usability. An SUS score >68 indicates an above-average usability [19]. The SUS score of all patients and the dentists and hygienists after each patient visit was calculated. A linear mixed effects model was used to examine factors that influence the SUS score of patients, including patient factors (demographic, socioeconomic, education, order of the patient seen in the eHygiene study, and time spent on eHygiene) while considering the nested random effects within practices and providers. Similar linear mixed effects models were used to examine factors that influence the SUS score of dentists and hygienists, including patient factors, DHCP factors, order of the patient seen in the eHygiene study, and time spent on eHygiene. The order of the patient being seen in the eHygiene study, with a cutoff whether before or after the seventh patient, was built in all the aforementioned models to assess whether the SUS score by DHCP is associated with a learning curve.

#### Dentist-Patient Communication

The Dentist-Patient Communication (DPC) scale was used to assess how well the patients understood the planned treatment and the quality of the communication between the patients and dentists using eHygiene. We used a modified questionnaire from a validated Doctor-Patient Communication questionnaire [20] that is often used in the medical field. We used a linear mixed effects model to examine the factors that influence the DPC score of patients while considering the nested random effects within practices and providers.

#### Qualitative Outcomes

We selected 15 individuals (5 patients, 5 dentists, and 5 hygienists) for 30-minute individual interviews virtually. These 15 individuals included those who rated above and below the average SUS score. The questions during the interview addressed the feedback, perceived challenges, and suggestions for improvement for the mDent eHygiene model. The interviews were standardized using interview guides (Multimedia Appendix 1), audio-recorded, transcribed, and analyzed for thematic content.

For the SELFIE session, the key patient tasks assessed were connecting cameras with the tablet, locating the photo-taking module, using a cheek retractor, taking front-view and posterior teeth photos, and ensuring that photographs were stored in the TeleDent platform. The user performance for key tasks was categorized into 3 levels: cosmetic (minor), critical (requiring assistance to proceed), and severe (resulting in significant delays or frustration). A study dentist also assessed the number of photographs and readable photographs using a photo assessment form.

#### Sample Size Consideration

We calculated the sample size based on the primary outcome, acceptance of mDent by patients that was calculated from patients’ SUS. Various studies [16,21,22] that have used the SUS scale to assess the usability of medical services or mHealth tools report a SUS score with a mean of 47.5-81.2 and an SD of 9.9-21.1. As patients in the eHygiene study were clustered by practice, we used a cluster randomized design for sample size calculation. Assuming that the SUS score difference between the patient-evaluated eHygiene model and other published mHealth tools has a mean of 8 and an SD of 10, a sample size of 72 patients from 12 practices (6 per practice) would achieve 90% power, with an α=.05. Consideration of sample size for primary and secondary outcomes was detailed in our protocol study [7].

### Ethics Approval

This study was peer-reviewed and funded by the National Institute of Dental and Craniofacial Research, United States. This study received single institutional review board approval from the University of Alabama at Birmingham (#300006506) and a local context review from the University of Rochester (#6077).

## Results

### Overview

The eHygiene study recruited 12 hygienists and 12 dentists from 12 US dental practices located in New York, New Jersey, Pennsylvania, and Connecticut. However, 3 hygienists and 3 dentists withdrew before receiving training because of limited time availability and concerns about the time needed for study activities. Moreover, 1 hygienist and 1 dentist received study training but did not start enrolling patients because of schedule conflicts. A total of 85 patients were recruited and enrolled by participating dental clinics; 2 patients withdrew before receiving intraoral images capture and virtual visits and 1 patient completed the office visit but not the virtual visit.

### Usability of eHygiene Among Patients

The demographic characteristics of patients are presented in Table 1. The mean age of the patients was 44.6 (SD 16.2, range 18-74) years. Approximately 70% (58/83) of these patients were female. Most of the patients were White (75/83, 90%), had private insurance (55/83, 66%), and resided in a suburban neighborhood (63/83, 76%). Approximately half of the patients (42/83, 51%) had a bachelor’s degree or higher. Interestingly, with all participants owning a smartphone, only 52% (43/83) had used medical care apps; however, none had ever used dental care apps. In addition, 41% (34/83) of the patients had previous experience taking photos of their teeth or mouth with their phones.

**Table 1:** Demographic characteristics of patients in eHygiene.

Parameters			Patients (n=83)
Age (years), mean (SD; range)			44.6 (16.2; 18-74)
Sex (female), n (%)			58 (70)
**Race, n (%)**			
	Black	7 (8)	
	White	75 (90)	
	Other	1 (1)	
Hispanic, n (%)			6 (7)
**Dental insurance, n (%)**			
	No insurance	17 (20)	
	Private	55 (66)	
	Government	6 (7)	
	Other	6 (7)	
**Education, n (%)**			
	High school	12 (14)	
	Some college or associate degree	29 (35)	
	Bachelor’s degree	25 (30)	
	Graduate degree	17 (20)	
**Community, n (%)**			
	Urban	8 (10)	
	Suburban	63 (76)	
	Rural	12 (14)	
**Household income (US $), n (%)**			
	25,001-50,000	15 (18)	
	50,001-100,000	24 (29)	
	>100,000	28 (34)	
	Prefer not to answer	16 (19)	
**Number of household members, n (%)**			
	1	11 (13)	
	2	28 (34)	
	≥3	44 (53)	
Owning a smartphone, n (%)			83 (100)
Use medical care apps, n (%)			43 (52)
Use dental care apps, n (%)			0 (0)
Previous experience of taking teeth or mouth photo, n (%)			34 (41)

**Figure 2:**
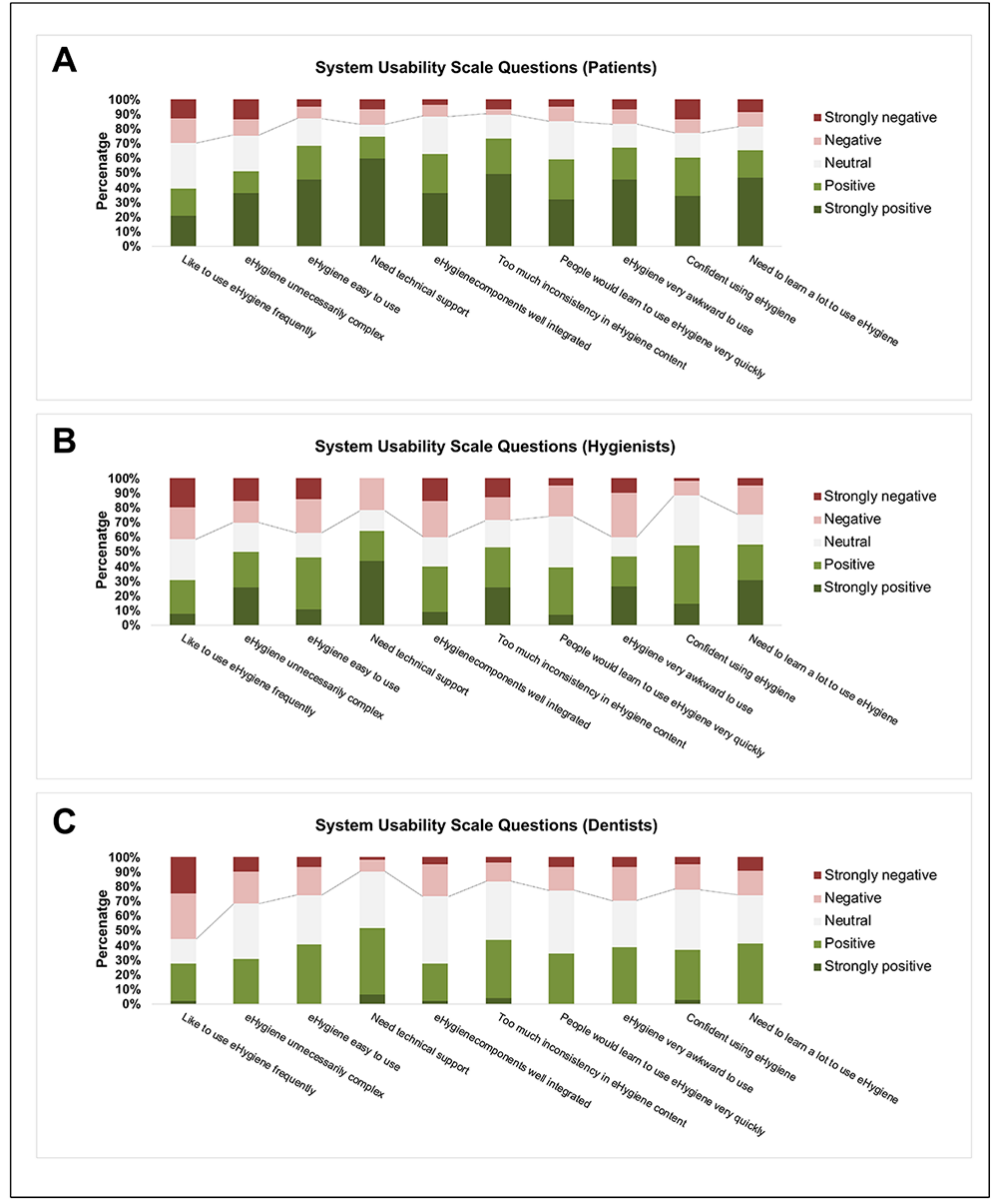
Acceptance of eHygiene among patients, hygienists, and dentists assessed by the System Usability Scale.

The eHygiene examination model was well accepted by patients, with a mean SUS score of 70 (SD 23.7). The patients’ SUS score was affected by the time spent on virtual visits and sex (Table S1 in Multimedia Appendix 2). Patients had low SUS scores when they spent more time on the virtual visit (*P*=.003). Females reported higher SUS scores (*P*=.03). The ratings of the individual items of the SUS are shown in Figure 2A. The response to each SUS item was converted to a scale from strongly negative to strongly positive. Across the 10 items, the responses from more than 60% (50/83) of the participants were neutral or positive (positive and strongly positive), indicating the well-perceived usability of the eHygiene model. For example, 60% (50/83) of the participants thought the eHygiene examination model was easy to use; 12% (14/83) of the participants felt that they needed technical support to use the eHygiene examination.

### Usability of eHygiene Among DHCP

**Figure 3:**
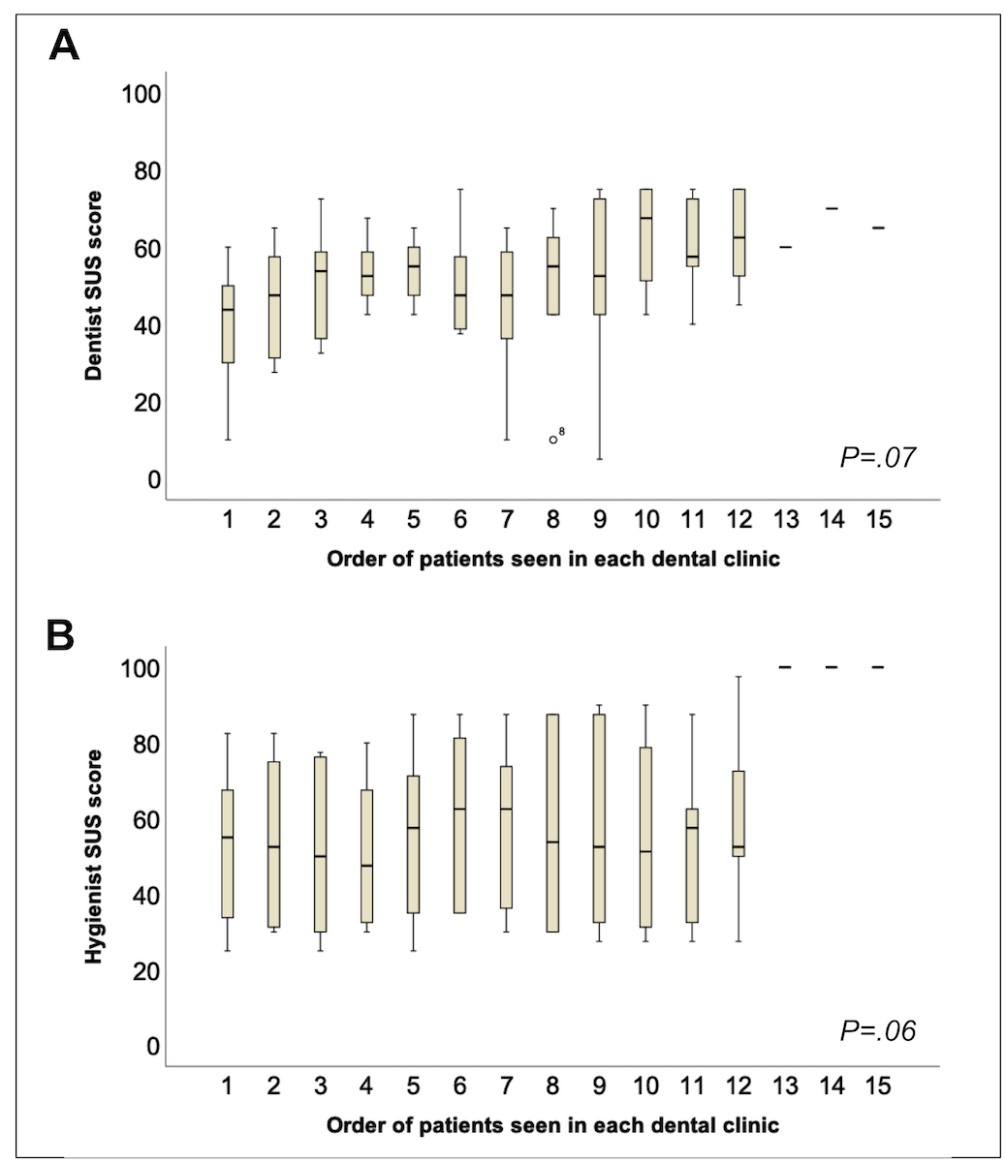
Learning curve of dentists and hygienists. Dentists' (A) and hygienists' (B) System Usability Scale (SUS) scores appear to be associated with a learning curve, with a higher SUS score given to patients who were seen after the first 7 patients in the study, after adjusting for dentist and hygienist demographics, patient characteristics, and time spent conducting hygiene examination visits (dentist: *P*=.07; hygienist: *P*=.06).

Overall, the race of 55% (5/9) of the dentists and 66% (6/9) of the hygienists was White. Half of the dentists and all hygienists were female. The average age was 56 (SD 7.0) years for the dentists and 42 (SD 12.5) years for the hygienists. The eHygiene model was moderately accepted by dentists (SUS score: mean 51.3, SD 15.9) and hygienists (SUS score: mean 57.1, SD 23.8). After examining each patient, every dentist or hygienist provided an SUS score for the eHygiene model, resulting in multiple SUS score values for each practitioner. The collected SUS scores from dentists and hygienists exhibited an association with a learning curve, wherein lower SUS scores were observed among patients treated initially, especially among the first 7 patients included in the study (dentist: *P*=.07; hygienist: *P*=.06; Figure 3). The ratings of the individual items of the SUS are shown in Figure 2B for the hygienists and Figure 2C for the dentists. Among the 2 items that are related to the learnability structure of the SUS [23], item 4 (I think that I would need the support of a technical person to be able to use eHygiene) and item 10 (I needed to learn a lot of things before I could get going with eHygiene), the results further suggested a learning curve related SUS learnability score among hygienists (Figure S1 in Multimedia Appendix 2) and dentists (Figure S2 in Multimedia Appendix 2). Furthermore, dentists reported lower SUS scores when spending more time on the virtual visit (*P*=.04). The results of the linear mixed effects regression model are detailed in Table S1 in Multimedia Appendix 2.

### DPC Using eHygiene

No statistical difference was observed between the current hygiene examination DPC (mean 58.5, SD 3.8) and the eHygiene examination DPC (mean 58.1, SD 5.97; *P*=.51). Both dentists and patients expressed that the oral findings and planned treatment were well understood by the patients (Table 2). The linear mixed effects regression model revealed that the current hygiene examination DPC was higher (*P*=.01) among patients residing in suburban communities after adjusting for other patient factors listed in Table S2 in Multimedia Appendix 2. Interestingly, the eHygiene examination of DPC was influenced by patient ethnicity. Hispanic patients had lower DPC scores than non-Hispanic patients (*P*=.01).

**Table 2:** Dentist and patient communication in the current hygiene model and the eHygiene model.

		Current hygiene examination	eHygiene examination	*P* value (*t* test)	
Dentist-Patient Communication score (patient evaluated, maximum score=60), mean (SD)		58.5 (3.8)	58.1 (5.97)	.51	
**How well do you think your patient understood what you explained about their oral health and the treatment you recommended? (** **Dentist evaluated, eHygiene n=83 patient visits** **), n (%)**					N/A^a^
	Very well	4 (44)	46 (55)		
	Fairly well	5 (56)	36 (43)		
	Fair	0 (0)	0 (0)		
	Poor	0 (0)	0 (0)		
	Very poor	0 (0)	0 (0)		

^a^N/A: not applicable.

### Patients Capable of Taking Diagnostic Intraoral Images

In the SELFIE session, all patients were able to complete the tasks with no or minor challenges (Table S3 in Multimedia Appendix 2). The longest time was spent on taking posterior teeth photographs (mean 4.7, SD 1.5 min). All other tasks, including capturing images of the front teeth and uploading photographs to TeleDent, took approximately 1 minute. No difference was found between patients and hygienists in terms of the total number and diagnostic qualities of the images taken (Table S4 in Multimedia Appendix 2). For instance, patients took an average of 26.5 photographs per person, whereas hygienists took an average of 33.3 photographs per person (*P*=.66). More than half of the photographs taken by the patients were diagnostic, indicating that the photographs were clear and included all anatomical structures of the teeth for diagnosis.

### Perception of the eHygiene Model by Patients and DHCP

Tables 3 and 4 indicate the quantitative perceptions of the current hygiene model and eHygiene model by patients and DHCP. The average time for taking intraoral images was 10 minutes. Most of the patients (76/83, 91%) reported that the photograph-taking procedure was comfortable. Dentists and patients consistently reported that the average time spent on a virtual visit was 6 minutes. Overall, the participating hygienists and the dentists thought the eHygiene model was suitable for the majority of patients with good oral health who did not have restorative or periodontal treatment in the past 1 year or more. Most of the DHCP indicated that eHygiene might not be suitable for patients with poor oral health who had ongoing untreated caries or periodontal disease, or for patients with urgent oral needs; for example, pain and orofacial swelling.

**Table 3:** Perspective on current (in-person) hygiene examination model by patients, hygienists, and dentists.

Survey questions			Patients’ perspective (n=83)		Hygienists’ perspective (n=9)		Dentists’ perspective (n=9)
**Time for in-person hygiene examination (min), n (%)**							
	1-4	23 (28)		3 (33)		1 (11)	
	5-10	55 (66)		6 (67)		8 (89)	
	>10	5 (6)		0 (0)		0 (0)	
**Time waiting for the dentist for examination (min), n (%)**							
	1-4	54 (65)		6 (67)		N/A^a^	
	5-10	26 (31)		3 (33)		N/A	
	>10	3 (4)		0 (0)		N/A	
**PPE^b^ changed when switching from seeing the chair side patient to a hygiene recall examination patient, n (%)**							
	Gloves	N/A		N/A		9 (100)	
	Surgical mask	N/A		N/A		5 (56)	
	N95 mask	N/A		N/A		0 (0)	
	Gown	N/A		N/A		1 (11)	
	Face shield or goggles	N/A		N/A		2 (22)	
	Bonnet	N/A		N/A		0 (0)	
**Charged PPE fees for hygiene recall examination patients since the COVID-19 pandemic started, n (%)**							
	No	N/A		N/A		7 (78)	
	1%-25%	N/A		N/A		0 (0)	
	26%-50%	N/A		N/A		1 (11)	
	51%-75%	N/A		N/A		0 (0)	
	76%-100%	N/A		N/A		1 (11)	
**Do you think it would be helpful if dental professionals used images on the computer or tablet to explain your oral health?, n (%)**							
	Yes, very helpful	40 (48)		N/A		N/A	
	Yes, to some degree	40 (48)		N/A		N/A	
	No, not helpful	0 (0)		N/A		N/A	
	I do not know	3 (4)		N/A		N/A	
**Do you routinely take intraoral images for your patients during hygiene visits?, n (%)**							
	Always	N/A		2 (22)		1 (11)	
	Very often	N/A		0 (0)		3 (33)	
	Sometimes	N/A		1 (11)		2 (22)	
	Rarely	N/A		1 (11)		3 (33)	
	Never	N/A		5 (56)		0 (0)	
**Do you use oral or teeth images to facilitate patient education or treatment planning?, n (%)**							
	Always	N/A		2 (22.2)		0 (0)	
	Very often	N/A		2 (22)		3 (33)	
	Sometimes	N/A		4 (44)		4 (44)	
	Rarely	N/A		0 (0)		2 (22)	
	Never	N/A		1 (11)		0 (0)	

^a^N/A: not applicable (the survey question was not answered by the group of participants).

^b^PPE: personal protective equipment.

**Table 4:** Perspective on the eHygiene examination model by patients, hygienists, and dentists^a^.

Survey questions and category		Patients’ perspective (n=83)	Hygienists’ perspective (n=83 patient visits, assessed by 9 hygienists)	Dentists’ perspective (n=83 patient visits, assessed by 9 dentists)
**Perceived patient’s comfortability when intraoral images were taken, n (%)**				
	Very comfortable	60 (72)	19 (23)	N/A^b^
	Somewhat comfortable	16 (19)	38 (46)	N/A
	Somewhat uncomfortable	5 (6)	18 (22)	N/A
	Very uncomfortable	0 (0)	5 (6)	N/A
	Extremely uncomfortable	—^c^	1 (1)	N/A
**Which types of patients do you think should be considered for the eHygiene virtual visits? (Choose all that apply), n (%)**				
	None	N/A	0 (0)	12 (14)
	Patients with good oral health who did not have restorative or periodontal treatment in the past 1+ year	N/A	62 (75)	70 (84)
	Patients with poor oral health who had ongoing untreated caries or periodontal disease.	N/A	9 (11)	0 (0)
	Patients with nonurgent oral diseases (eg, caries, periodontal pocket deeper than 4 mm, etc) identified by hygienists during cleaning.	N/A	32 (39)	2 (2)
	Patients with urgent oral needs, for example, pain, orofacial swelling, etc.	N/A	7 (8)	1 (1)
	Patients with oral mucosal lesions identified by hygienists during cleaning.	N/A	23 (28)	0 (0)
	All patients	N/A	7 (8)	0 (0)

^a^The eHygiene model was well accepted by patients (System Usability Scale score: mean 70.0, SD 23.7) and moderately accepted by hygienists (System Usability Scale score: mean 57.1, SD 23.8) and dentists (System Usability Scale score: mean 51.3, SD 15.9). Time spent (min) on taking intraoral images by hygienists (mean 9.8, SD 5.7; range 1-25). Time spent (min) conducting the eHygiene virtual visit by patients (mean 5.9, SD 5.3; range 1-30) and dentists (mean 6.4, SD 5.1; range 2-40). Of the 83 patient visits, eHygiene visits during work hours by patients was 41 (49%) and for dentists 29 (35%).

^b^N/A: not applicable (the survey question was not applicable to the specific group of participants).

^c^Not available.

The qualitative analysis further indicates 8 thematic patterns of patients’ and DHCP’s perspectives on the eHygiene model. The first 4 are the foreseen benefits of eHygiene, which include an overall positive experience for patients (theme 1), eHygiene enables effective communication to the patient about oral health (theme 2), eHygiene saving resources (theme 3), and eHygiene has the potential to improve oral health care services (theme 4). However, patients and DHCP also talked about 4 limitations of eHygiene: the current eHygiene model does not provide all necessary oral health data needed to make comprehensive evaluations (theme 5), extra time is needed because of the technology-related learning curve and technical issues (theme 6), eHygiene lacks interpersonal interaction (theme 7), and selectivity in eHygiene use (theme 8). Representative quotes are listed in Table S5 in Multimedia Appendix 2.

In addition, the participating dentists and hygienists raised concerns regarding monetary and reimbursement issues. Specifically, they pointed out that the time required for intraoral photo capture and virtual dental visits was not traditionally reimbursed under the current dental fee schedule. As such, they emphasized the need for recognition of these services by insurance payers or patients to facilitate adoption of the service. Addressing these concerns and finding viable reimbursement options may help to increase the adoption and sustainability of the mDent model.

## Discussion

### Principal Findings

The eHygiene study had multiple strengths. First, we were successful in engaging patients and nondentist professionals in the dental office in capturing essential oral health data (intraoral images and x-rays) and conducting virtual dental hygiene examinations (mDent model). This inclusive engagement is novel and potentially transformative to dental practice. The use of smartphones and mobile devices to take photos of the mouth and teeth and conduct oral disease screening has been recently reported [6,24,25]; however, the feasibility of engaging dental hygienists and dental patients to obtain intraoral images using an intraoral camera has not been previously assessed. Second, transforming the traditional one-to-one visual and tactile dentist examination to an eHygiene visit requires a team effort from several stakeholders, including patients, dental hygienists, and dentists. The team efforts could lead to better DHCP-patient communication and a better understanding of and compliance with this approach to dental treatment. Third, integrating the mHealth concept into dentistry to achieve population-wide oral health screening and monitoring is extremely innovative and offers a vehicle to promote patient-engaged oral health education and early detection of patient-driven oral diseases. Fourth, the eHygiene model is a novel way of preserving PPEs during the COVID-19 pandemic and other respiratory transmissible disease outbreaks. In addition to the abovementioned strengths, the eHygiene study has the following limitations: (1) a limited number of patients participated in the SELFIE session, which limits the generalization of the study results from the SELFIE session; and (2) the study was conducted in dental clinics residing in suburban areas with most of the participating patients having private dental insurance. Therefore, the study results cannot be generalized to the underserved population or dental office in the community setting.

During the early time of the COVID-19 outbreak, the American Dental Association issued recommendations for their fellow dentists to provide care to emergency patients only. According to the American Dental Association, as of 2019, general dentists in the United States are delivering 564 million patient visits per year [26]. Notably, 316 million (56%) of these 564 million patient visits are examination visits that are often not linked to definite treatment delivery at the same visit. Under the circumstances that routine office dental visits were reduced during the COVID-19 outbreak, there were anecdotal reports that some patients and dentists chose to use intraoral images taken by patients to assess urgent dental situations; for example, fractured tooth and facial swelling. This new phenomenon of patient-dentist communication provides an opportunity for a new way of delivering dental service, mDentistry, that could transform community dentistry.

Although the eHygiene model was well accepted by patients, the eHygiene model was conservatively accepted by dentists and dental hygienists. On the basis of the study findings, it appears that the SUS scores of both dentists and hygienists may correlate with a learning curve. Specifically, higher SUS scores were observed in visits that occurred after the first 7 patients were seen in the study. This suggests that with experience and practice, dental professionals may become more comfortable and proficient in using the system, leading to increased user satisfaction. Furthermore, SUS measures both usability (8 items) and learnability (2 items: item 4 and item 10) [23]. The subanalysis of the SUS learnability of the dentists and hygienists confirmed a potential learning curve associated with the eHygiene model. This indicates that additional training may be necessary before DHCP implement the eHygiene model in their daily clinical practice. Another intriguing point raised by dentists and hygienists was monetary and reimbursement concern, which might also be associated with the SUS score given by DHCP. Although some hygienists expressed that patients would save time and money by using eHygiene, others expressed concerns about losing revenue or not being appropriately reimbursed for eHygiene services because of the extended appointment time. Insurance reimbursement for eHygiene work was considered crucial to support the sustainability of the mDentistry eHygiene model.

In addition, the eHygiene study used SUS to assess acceptance, and the other system that has been used to assess individuals’ acceptance of technology service is the UTAUT (Unified Theory of Acceptance and Use of Technology) [27-29]. Although SUS is a widely used questionnaire-based tool for measuring the perceived usability of a system or product; the UTAUT, on the other hand, is a theoretical model that seeks to explain and predict user acceptance and use of technology [30-32]. The UTAUT is useful for predicting and understanding user acceptance and use of technology in various contexts and can be used to identify potential barriers to technology adoption and use [33]. Both frameworks can be used to improve the design and development of technology-based products and systems.

The mDentistry eHygiene model offers a complementary modality for oral health data collection, which would be particularly useful during an infectious disease outbreak. The fact that patients can capture critical oral health data in a home setting could facilitate dental treatment triage and oral health self-monitoring and potentially trigger oral health–promoting behaviors. This hypothesis of self-monitoring associated with oral health behavior changes could be tested in a clinical trial that assessed the impact of mDent on oral health promotion at a population base.

Notably, mDentistry is at the intersection of incorporating artificial intelligence (AI) into dentistry. AI represents an emerging adjunct to caries screening and risk management, building upon (1) reliability of teledentistry that uses intraoral images and live videos to make diagnostic decisions [9,13,14,34] and (2) rapid advancement of mHealth tools use by all-age Americans [35,36]. Recently, AI had been tested in detecting caries and oral pathologies on dental x-rays [37,38]. Our team has developed a smartphone app, AICaries, that uses AI-powered technology to detect caries on photographs of teeth taken via smartphone [39-41]. As AI is currently used to aid imaging recognition to improve disease diagnosis in many medical fields, including oncology, ophthalmology, and radiology [42-45], AI has the full potential to be developed in dentistry for remote caries detection and caries risk management for underserved patients with limited access to oral health care. Future clinical service transformation should leverage the convenience provided by mDentistry and the robust disease screening powered by AI technology to improve oral health early detection and prevention at a broad population base. A population-wide intraoral images and x-rays database is urgently needed to be developed to facilitate oral disease screening automation in the community.

### Conclusions

The eHygiene study results informed the process and usability of the mDentistry eHygiene model and showed promise for conducting virtual dental examinations and empowering patients with mHealth tools. The eHygiene model was well accepted by patients and moderately accepted by dentists and hygienists. Changes in reimbursement could accelerate its adoption. In addition, the complementary modality for oral health data collection and examination in dental offices provided by eHygiene would be beneficial during an infectious disease outbreak.

## Appendix

Multimedia Appendix 1 Interview guide.

Multimedia Appendix 2 Supplementary material.

## Abbreviations

AI: artificial intelligence
DHCP: dental health care personnel
DPC: Dentist-Patient Communication
mHealth: mobile health
PBRN: Practice-Based Research Network
PPE: personal protective equipment
SUS: System Usability Scale
UTAUT: Unified Theory of Acceptance and Use of Technology
