# Supplementary material for: Acceptance and Usability of an Innovative mDentistry eHygiene Model Amid the COVID-19 Pandemic Within the US National Dental Practice-Based Research Network: Mixed Methods Study
Source: JMIR Hum Factors. 2023 Aug 18;10:e45418. doi: 10.2196/45418 (PMC10474507; doi:10.2196/45418)
Supplement: Multimedia Appendix 2 [file humanfactors_v10i1e45418_app2.docx]

**Table S1 Factors associated with SUS scoring (Linear Mixed Effects Regression Model)**

|  | **Patients** | | | **Dentists** | | | | **Hygienists** | | |
| --- | --- | --- | --- | --- | --- | --- | --- | --- | --- | --- |
| **Covariates** | **E** | **SE** | **P** | **E** | **SE** | **P** | **E** | | **SE** | **P** |
| (Constant) | 71.74 | 14.8 | 0.002 | 84.68 | 142.18 | 0.58 | 69.23 | | 31.51 | 0.08 |
| Patient order in eHygiene study (first 7 patients/after 7 patients) | 8.46 | 5.44 | 0.13 | -5.32 | 2.84 | 0.07 | -4.50 | | 2.31 | 0.06 |
| Patient gender (Female/Male) | 12.64 | 5.79 | 0.03 | **-** | **-** | **-** | **-** | | **-** | **-** |
| Patient age | -0.17 | 0.15 | 0.28 | **-** | **-** | **-** | **-** | | **-** | **-** |
| Patient ethnicity (Hispanic/Non-Hispanic) | 6.12 | 7.36 | 0.41 | **-** | **-** | **-** | **-** | | **-** | **-** |
| Patient Race (white/Other) | -2.19 | 9.32 | 0.81 | **-** | **-** | **-** | **-** | | **-** | **-** |
| Hygienist Age | **-** | **-** | **-** | **-** | **-** | **-** | 0.002 | | 0.78 | 0.99 |
| Hygienist Race (White/Other) | **-** | **-** | **-** | **-** | **-** | **-** | -3.57 | | 20.66 | 0.86 |
| Dentist gender (Female/Male) | **-** | **-** | **-** | -3.74 | 21.22 | 0.86 | **-** | | **-** | **-** |
| Dentist age | **-** | **-** | **-** | -0.37 | 2.65 | 0.89 | **-** | | **-** | **-** |
| Dentist Race (White/Other) | **-** | **-** | **-** | -5.14 | 28.95 | 0.86 | **-** | | **-** | **-** |
| Patient Dental Insurance (government support/non-Government support) | 1.16 | 10.25 | 0.91 | -1.32 | 5.03 | 0.80 | 2.32 | | 4.23 | 0.58 |
| Patient education (≥college/ <college) | -1.42 | 4.94 | 0.77 | -2.17 | 2.66 | 0.42 | -0.81 | | 2.22 | 0.72 |
| Patient Residing in suburban community (Y/N) | 5.42 | 6.12 | 0.38 | 0.58 | 3.25 | 0.86 | -3.60 | | 2.66 | 0.87 |
| Patient Income level >50k (Y/N) | 7.97 | 7.15 | 0.27 | 0.28 | 3.58 | 0.94 | -0.50 | | 3.10 | 0.87 |
| Time of taking intraoral photos | -0.88 | 0.47 | 0.07 | - | - | - | -0.48 | | 0.51 | 0.35 |
| Duration of virtual visit (reported by dentist, minutes) | **-** | **-** | **-** | -0.63 | 0.29 | 0.04 | - | | - | - |
| Duration of virtual visit (reported by patient, minutes) | -1.45 | 0.48 | 0.003 | - | - | - | - | | - | - |

SUS: System Usability Score

E: Estimate

SE: Std. Error

P: P value

**Table S2 Factors associated with DPC scoring (Linear Mixed Effects Regression Model)**

|  | **DPC**  **(Current hygiene)** | | | **DPC**  **(eHygiene)** | | |
| --- | --- | --- | --- | --- | --- | --- |
| **Covariates** | **E** | **SE** | **P** | **E** | **SE** | **P** |
| (Constant) | 58.35 | 2.31 | <0.001 | 47.23 | 13.24 | 0.01 |
| Patient order in eHygiene study (among the first 7 in each clinic) | 0.31 | 0.83 | 0.71 | 2.17 | 1.51 | 0.16 |
| Patient gender (female) | -1.51 | 0.93 | 0.11 | -1.02 | 1.65 | 0.54 |
| Patient age | <0.001 | 0.02 | 0.97 | 0.04 | 0.04 | 0.38 |
| Patient ethnicity (Hispanic) | 0.13 | 1.15 | 0.91 | -5.62 | 1.98 | 0.01 |
| Patient Race (white) | -2.53 | 1.46 | 0.09 | -2.42 | 2.63 | 0.36 |
| Patient Dental Insurance (government support) | 0.96 | 1.54 | 0.54 | 1.37 | 2.81 | 0.63 |
| Patient education (≥college) | 1.13 | 0.78 | 0.15 | 0.53 | 1.37 | 0.70 |
| Patient Residing in suburban community | 2.54 | 0.94 | 0.01 | -0.36 | 1.83 | 0.85 |
| Patient Income level >50k | 0.94 | 1.15 | 0.42 | -1.74 | 1.98 | 0.38 |
| Time of taking intraoral photos | **-** | **-** | **-** | 0.03 | 0.16 | 0.83 |
| Duration of virtual visit (reported by patient, minutes) | **-** | **-** | **-** | -0.08 | 0.13 | 0.54 |
| DPC score for current (in-person) hygiene model | **-** | **-** | **-** | 0.22 | 0.21 | 0.31 |

DPC: Dentist Patient Communication

E: Estimate

SE: Std. Error

P: P value

**Table S3 Time spent and challenges encountered during Selfie (n=4)**

| **Tasks during Selfie** | **Time (min)** | **Challenges (Minor)** |
| --- | --- | --- |
| Connecting device | 0.1 | 0 |
| Locate app to take intraoral photos | 1.3±0.6 | 2 (66.7%) |
| Able to use intraoral camera | 0.2±0.2 | 1 (33.3%) |
| Take front teeth photos | 0.3±0.2 | 0 |
| Take posterior teeth photos | 4.7±1.5 | 1 (33.3%) |
| Upload photos to TeleDent | 1.4±1.0 | 2 (66.7%) |

Note: only minor challenges were encountered.

**Table S4 Capability of taking teeth photos by patients**

| **Number of photos** | **Selfie Patients**  **(n=4)** | **Hygienists**  **(n=4)** | **p-value** |
| --- | --- | --- | --- |
| All teeth photos | 26.5±18.3 | 33.3±18.9 | 0.66 |
| All diagnostic teeth photos | 19.5±11.3 | 30.7±18.2 | 0.36 |
| Front view photos | 2.5±1.3 | 1.3±0.6 | 0.21 |
| Diagnostic front view photos | 1.8±1.8 | 1.0±1.0 | 0.53 |

**Figure S1. Learnability structure of SUS of hygienists**

The Item 4 (A) and Item 10 (B) of SUS scores for hygienists appear to be associated with a learning curving.

**Figure S2. Learnability structure of SUS of Dentists**

The Item 4 (A) and Item 10 (B) of SUS scores for Dentists appear to be associated with a learning curving.
